# Breastfeeding behavior is not associated with health literacy: evidence from the German KUNO-Kids birth cohort study

**DOI:** 10.1007/s00404-021-06038-2

**Published:** 2021-04-27

**Authors:** Teresa M. Graus, Susanne Brandstetter, Birgit Seelbach-Göbel, Michael Melter, Michael Kabesch, Christian Apfelbacher, Sara Fill Malfertheiner, the KUNO-Kids study group, A. Ambrosch, P. Arndt, A. Baessler, M. Berneburg, St. Böse-O’Reilly, R. Brunner, Wolfgang Buchalla, A. Franke, S. Häusler, I. Heid, C. Herr, W. Högler, S. Kerzel, M. Koller, M. Leitzmann, D. Rothfuß, W. Rösch, B. Schaub, B. H. F. Weber, St. Weidinger, S. Wellmann

**Affiliations:** 1 University Department of Gynecology and Obstetrics, Hospital St. Hedwig of the Order of St. John, University Medical Center Regensburg, Steinmetzstr., 1-3, 93049 Regensburg, Germany; 2 University Children’s Hospital Regensburg (KUNO), Hospital St. Hedwig of the Order of St. John, University of Regensburg, Steinmetzstr., 1-3, 93049 Regensburg, Germany; 3 The Research and Development Campus Regensburg (WECARE), Hospital St. Hedwig of the Order of St. John, University of Regensburg, Regensburg, Germany; 4 Institute of Social Medicine and Health Systems Research, Otto Von Guericke University Magdeburg, Leipziger Str. 44, 39120 Magdeburg, Germany

**Keywords:** Health literacy, Health care, Breastfeeding, Breastfeeding promotion

## Abstract

**Purpose:**

Despite the health benefits of full breastfeeding for both infants and mothers, less than 50% of mothers in Germany practice this method for at least 4 months after childbirth. Because of the growing importance of health literacy to improve public health, we investigated the role of maternal health literacy in breastfeeding behavior.

**Methods:**

We analyzed the data of 1172 mother–child dyads of the KUNO-Kids health study of the University Children’s and Maternity Hospital Regensburg. Maternal health literacy was assessed with the HLS-EU-Q47 questionnaire (sub-index health care) up to 48 h after childbirth. Outcome was analyzed 6 months after childbirth and categorized into full breastfeeding for less than 4 months or for at least 4 months. The association between breastfeeding and maternal health literacy was calculated with univariable and multivariable logistic regression analyses.

**Results:**

38.8% of mothers showed inadequate or limited health literacy. 75.9% of mothers had fully breastfed their child for at least 4 months. Univariable logistic regression analysis showed that health literacy and full breastfeeding for at least 4 months were not associated (OR = 0.995 [CI 0.977–1.015], *p* = 0.60). After adjusting for all potentially confounding variables with a significant association (*p* ≤ 0.05) on both health literacy and breastfeeding, the multivariable model showed no association between health literacy and breastfeeding (OR = 0.984 [CI 0.963–1.007], *p* = 0.170).

**Conclusion:**

Surprisingly, we found no association between health literacy and breastfeeding behavior in our study. Therefore, future research with comparable measurements of health literacy and breastfeeding is required to validate this result and to identify reasons for early breastfeeding cessation.

## Introduction

Breastfeeding protects children from infections, improves cognitive development, and reduces the development of obesity, chronic diseases and even some types of cancer [1–5]. On top of improving a child’s health, various positive gynecological outcomes have been described for mothers: a lower risk of developing diabetes, cardiovascular diseases, as well as ovarian and premenopausal breast cancer [6]. Nevertheless, up to 54% of children in Germany are not fully breastfed for at least 4 months as recommended by the national commission for breastfeeding in Germany [7–9].

Multiple factors, such as breastfeeding problems or the educational status of the mother, may influence women’s decisions not to breastfeed, which presents a potential risk to the development and health of the infants [8, 10]. Besides other potential risk factors for premature weaning or the decision to never start breastfeeding at all, several studies have found that mothers with low health literacy (HL) are less likely to breastfeed their infants [11–15].

However, Mirjalili et al. did not find any association between HL and breastfeeding behavior in their Iranian study cohort [15]. Thus, scientific evidence on the association between HL and breastfeeding is contradictory.

In their 2030 Agenda for Sustainable Development established in November 2016, the Global Conference on Health Promotion emphasized the importance of facilitating the development of HL as “a critical determinant of health” [16]. In fact, according to the German Health Update study of 2013 (GEDA 2013s) with 4845 participants, 44.5% of German female habitants of childbearing age (between 18 and 39 years) have limited HL, which suggests that they have problems to find, understand, appraise, and apply information on health issues [17]. Limited HL impedes people’s capability “to make judgments and take decisions in everyday life concerning healthcare, disease prevention, and health promotion to maintain or improve quality of life during the life course” [18]. Since its introduction in the 1970s, the concept of HL has gained importance worldwide. Thus, after the identification of this extensive HL deficit in Germany, a national action plan was launched in February 2018 to enhance HL in the German population [19, 20].

Women with a higher level of HL are likely to know more about the positive effects of breastfeeding, may have better strategies to solve problems, or may be more capable to reflect on their own behavior.

In view of the above-mentioned findings and the fact that more than 50% of the German population have limited HL, we investigated if the HL of mothers influence their breastfeeding behavior [21].

As far as we are aware, this is the first European study to investigate the association between HL and breastfeeding behavior. The analysis was based on data obtained from the large birth cohort study KUNO-Kids from Eastern Bavaria, Germany, to improve medical care in this patient population [22]. This investigation is highly relevant, particularly in view of the knowledge that adequate breastfeeding is one of the best strategies for disease prevention in both mothers and infants.

## Methods

### Study design and setting

We analyzed the data of the prospective multi-purpose KUNO-Kids health study on women who give birth in the Clinic St. Hedwig in Regensburg, Eastern Bavaria, Germany [22]. The Clinic St. Hedwig is a mother and child hospital affiliated with the University Medical Center Regensburg that counts approximately 3300 births per year [22].

The KUNO-Kids cohort comprises mother–child dyads who are prospectively studied by the following means: in hospital, mothers are interviewed and have to fill in questionnaires within 2 to 3 days after childbirth. Afterwards, a follow-up questionnaire is sent out 4 weeks and 6 months after childbirth and in every year until the child reaches 18 years of life.

### Participants and data collection

As reported previously, mothers were asked to take part in the study within 48 h after giving birth [22]. They were given 1 day for consideration. Inclusion criteria for the KUNO-Kids health study were signed informed written consent of the mother, legal age, sufficient knowledge of the German language, and not participating in the study with another child.

The data for this analysis were obtained from personal interviews conducted by a study assistant at the clinic within 2 days after childbirth and from follow-up questionnaires sent out 4 weeks and 6 months after childbirth. Data of children born between June 2015 and September 2018 were collected. Table 1 shows the distribution of the received questionnaires for the various points in time.

**Table 1:** Follow-up rates of the study period

	*N*	% of total *N* = 2657
Baseline received	2657	100.0
4-week follow-ups received	1647	62.0
6-months follow-ups received	1384	52.1
Dyads with all three data sets	1228	46.2

Exclusion criteria of this analysis were multiple birth and the intake of medicine during pregnancy that is not recommended for being taken while breastfeeding. Furthermore, we excluded women with medically treated mastitis, assuming that they ceased breastfeeding because of this complication. Table 2 shows the number of women included after controlling for exclusion criteria.

**Table 2:** Number of mother–child dyads meeting the inclusion criteria

	*N*	% of *N* = 1228^a^
No professionally diagnosed mastitis	1173	95.5
No drug intake incompatible with breastfeeding	1227	99.9
Total number of includable cases for this analysis	1172	95.4

^a^Number of mother–child dyads with all three data sets

### Variables and measurement

#### Health literacy

HL as the exposure of interest is investigated by means of the sub-index health care of the standardized and validated HLS-EU-Q47 questionnaire. This questionnaire is measured as a multidimensional 12-cell matrix with questions combining each of the four key processes of accessing, understanding, appraising, and applying health-related information [23]. Each of the 16 questions on health care, investigated by means of the interview could be answered with “very difficult”, “rather difficult”, “rather easy”, or “very easy” on a four-point Likert-type scale. The interview included questions, such as “How difficult/easy is it for you to find information on symptoms you have developed?” or “How difficult/easy is it for you to find out if information on your illness in the media is reliable?” The health care HL index results in a score ranging between 0 and 50. For the sample description, the scores were categorized into four levels: “inadequate” (0–25), “problematic” (> 25–33), “sufficient” (> 33–42) and “excellent” (> 42–50) [23, 24]. Scores in the regression analyses were used as continuous variables.

#### Full breastfeeding

The investigation’s outcome was the rate of full breastfeeding 4 months after childbirth that was investigated by means of the interview’s question “Do you breastfeed your child?” and the 6-month follow-up question “How long did you breastfeed your child without giving any complementary food?”. Full breastfeeding in this context means, that the child only received breast milk but no formula or complementary food. We dichotomized full breastfeeding into less than 4 months and at least 4 months after childbirth.

#### Confounders

The following variables were considered as potential confounders.

Age, parity, and the marital status of the mother were assessed by means of direct questions during the interview.

The educational level was categorized according to the “Comparative Analysis of Social Mobility in Industrial Nations (CASMIN)” classification in the latest version, including education and vocational education, in “low” (CASMIN 1), “moderate” (CASMIN 2) and “high” (CASMIN 3) [25, 26].

Migration background was indexed on the basis of the country of birth of the mother or her parents outside Germany.

Maternal smoking was documented, if the mother had smoked or had consumed alternative smoking products during pregnancy or smoked regularly after childbirth, and this aspect was investigated within the 4-week follow-up.

The mother’s health-related quality of life was quantified by means of the Short Form Health Survey Questionnaire (SF-12) [27]. Quality of life was measured using the two subscales of the questionnaire, i.e. physical health (PSC) and mental health (MCS). Each subscale may yield values between 0 and 100 [28].

The body mass index in kg/m^2^ prior to pregnancy was calculated in accordance with the values for height and weight given in the personal interview. Physical activity prior to pregnancy was categorized according to the recommendation of the World Health Organization of not less than 2.5 h of physical activity per week and during pregnancy according to the recommendation of the German Association for Sports Medicine and Prevention of 1–2 h of physical activity per week [29, 30]. Hence, the information about physical activity before and during pregnancy obtained in the interview was scaled in “less than recommended”‚ “as recommended” or “more than recommended”. The 4-week follow-up also included questions about alcohol consumption before and during pregnancy as well as after childbirth. Alcohol consumption of the mother was evaluated according to the number of standard alcoholic drinks per week and was categorized as risky if the amount of alcoholic drinks was six or more per week in the year prior to pregnancy or if the mother had consumed any alcohol during pregnancy and lactation [31].

The 6-month follow-up included questions on the mother’s occupation between giving birth and 6 months after childbirth.

A German version of the MacArthur Scale was used to measure the mother’s subjective social status at the 4-week follow-up. Using an imaginary ladder with 10 rungs, the participants had to rate their personal social status as compared to their community members between 0 (least money, lowest education, and worst jobs) and 10 (most money, highest education, and best jobs) [32].

Midwife service was assessed at the 6-month follow-up.

### Statistical analysis

To describe the sample’s socio-demographic data, we calculated means with standard deviations for continuous data and frequencies with percentages for categorical data. The duration of full breastfeeding in weeks is presented using mean with standard deviation, and the outcome variable full breastfeeding for at least 4 months is presented using frequency with percentage.

Univariable logistic regression analysis was used to evaluate the association between the HL index as a metric scale and full breastfeeding for less than 4 months vs. for at least 4 months. Additionally, multivariable logistic regression analysis was used to control for confounders that showed a statistically significant association (*p* ≤ 0.050) in univariable analysis for both the HL index and breastfeeding.

Statistical analyses were done with IBM SPSS.23.

## Results

This analysis included 2657 mother–child dyads. All participants in the first assessment were invited to participate in the follow-up assessments. Therefore, longitudinal analyses after 6 months were possible for 1228 dyads (Table 1). Finally, 1172 mother–child dyads met the inclusion criteria (Table 2).

38.8% of mothers showed inadequate or limited HL, whereas 18.5% had an excellent HL level. 75.9% of mothers fully breastfed their child for at least 4 months and the mean duration of full breastfeeding in the study population was 16.4 weeks. The characteristics of the study population are shown in Table 3.

**Table 3:** Socio-demographic characteristics of the sample

	*N*^a^ (total *N* = 1172)	Value
Duration of pregnancy (weeks) *M* (SD)	1164	39.58 (1.58)
Preterm births (≤ 36 + 6 gestational weeks, *M* = 35.37) *N* (%)		68 (5.84)
Infant’s sex (female) *N* (%)	1172	588 (50.2)
Maternal age (years) *M* (SD)	1161	34.56 (4.26)
Birth mode (cesarean section) *N* (%)	1172	319 (27.2)
Parity (two or more children) *N* (%)	1164	474 (40.7)
Marital status of the mother	1157	
Married *N* (%)		948 (81.9)
Unmarried but living together with a partner *N* (%)		193 (16.7)
Unmarried without a partner, divorced, or widowed *N* (%)		16 (1.4)
Maternal education	1145	
CASMIN 1 [elementary education] *N* (%)		80 (7.0)
CASMIN 2 [intermediate education] *N* (%)		512 (44.7)
CASMIN 3 [tertiary education] *N* (%)		553 (48.3)
Maternal migration background *N* (%)	1100	180 (16.4)
Maternal smoking *N* (%)	1159	43 (3.7)
Health literacy (HLS-EU sub-index health care) *M* (SD)	1143	35.30 (7.21)
Inadequate (0–25^b^) *N* (%)		87 (7.6)
Limited (> 25–33^b^) *N* (%)		357 (31.2)
Sufficient (> 33–42^b^) *N* (%)		488 (42.7)
Excellent *N* (> 42–50^b^) (%)		211 (18.5)
Breastfeeding at hospital discharge *N* (%)	1158	1018 (87.9)
Full breastfeeding for at least 4 months *N* (%)	1079	819 (75.9)
Duration of full breastfeeding^c^ (weeks) *M* (SD)		16.40 (9.27)

*N* number of women’s answers regarding the individual item, *M* mean value, *SD *standard deviation

^a^The difference between the total numbers is due to missing data for this specific item

^b^Score ranges of the HL index regarding the specific level

^c^As assessed in the 6-months questionnaire

Univariable logistic regression analysis showed that the HL index and full breastfeeding for at least 4 months were not significantly associated [odds ratio (OR): 0.995, 95% confidence interval (95% CI) [0.977–1.015], *p* = 0.600, *N* = 1094]. This finding did not change after adjusting for parity, marital status, maternal education, SF-12 MCS, physical activity during pregnancy, subjective social status, and midwife service (OR: 0.986 CI [0.964–1.008], *p* = 0.218, *N* = 964). The discrepancy between the total numbers stated is due to missing data for items that were relevant for the logistic regression analyses.

## Discussion

Surprisingly, we did not find any positive association between the HL level of mothers and their breastfeeding behavior when considering breastfeeding for at least 4 months versus less than 4 months after childbirth. This finding is not in line with the extant literature on this topic.

Comparability to other studies is limited because of the widely varying measurement of HL and the inconsistent definitions of the outcome breastfeeding. Yin et al., Stafford et al., and Kaufman et al. quantified HL by means of questionnaires that exclusively measured functional HL in their study populations: Short Test of Functional Health Literacy in Adults (S-TOFHLA) [Yin and Stafford] or Rapid Estimates of Adult Literacy in Medicine (REALM) [Kaufman]. REALM measures the reading and pronunciation ability of medical words and texts only, whereas S-TOFLA also integrates comprehension of the terms [11, 12, 14, 33]. All authors found positive correlations between HL and breastfeeding behavior, although the outcome breastfeeding was assessed differently in each study: feeding of a higher proportion of breastmilk than formula [Yin], a higher rate of exclusive breastfeeding shortly after birth [Stafford], and exclusive breastfeeding for more than 2 months [Kaufman] [11, 12, 14]. Vila-Candel et al. found no statistical significant association between functional HL measured by newest vital sign (NVS) and exclusive breastfeeding for more than 4 months [34].

Using the Iranian questionnaire HELIA (Health Literacy Instrument for Adults), Hosseini et al. and Mirjalili et al. assessed HL more comprehensively and included accessing, understanding, evaluating, and applying of health-related information [13, 15, 35, 36]. Inconsistent assessment of breastfeeding should also be noticed: breastfeeding duration in general [Hosseini] and breastfeeding patterns that aim at the proportion of breastmilk in contrast to formula feeding [Mirjalili] [13, 15]. Only the results of Mirjalili et al. were in line with ours, as these authors did also not find any statistically significant association between HL and breastfeeding [15].

We chose a subscale of the HLS-EU, the most extensive instrument established so far, which assesses “all areas of health literacy [in combination with] social determinants of health”, such as social support or accessibility to health care services [37]. It measures not only individual skills but also “the self-experienced and self-rated relation or fit of personal competencies and situational demands/complexity” [38, 39]. Jordan et al. and Freedman et al. emphasized that additional environmental influences, such as social, socioeconomic, or healthcare-related issues, may mitigate the ability to deal with health information [40, 41]. It is remarkable that our study’s broader assessment of HL led to a different result than solely assessing (functional) HL.

A mother’s decision to breastfeed could be based on socio-cultural reasons or on model learning from their relatives or peers, which may also predominate the decision on the duration of breastfeeding. Kohlhuber et al., for example, identified the attitudes of the partner and the maternal grandmother towards breastfeeding as an influencing factor for the duration of breastfeeding [42].

Moreover, the recent SuSe II study has shown that health education on breastfeeding is more and more implemented in birth clinics throughout Germany, for example, by means of prenatal classes and midwife support [43]. Hence, mothers may develop better breastfeeding behavior because of this specific support, independent of individual HL levels.

Nevertheless, to improve breastfeeding rates, it seems relevant to further strengthen specifically tailored programs for breastfeeding promotion, such as targeted information about breastfeeding, breastfeeding instructions, and incorporation in the social environment.

### Strengths and limitations

The initial breastfeeding rate of 87.9% found in our study was rather similar to the rate of 87.3% described in the German KiGGS study (study on the health of children and adolescents in Germany) (birth cohort 2013/2014) [9]. The full breastfeeding rate of 75.9% for 4 months after childbirth in our study was much higher than the rate of 46.0% in the KIGGs study (birth cohort 2009–2014) and the rate of 60.1% in the SuSe II study (birth cohort January–March 2018) [9, 43]. The high breastfeeding rate in our sample may be due to the inclusion of mother–child dyads in a prospective study design. In contrast to cross-sectional analyses of breastfeeding rates in studies, such as KIGGs or SuSe II, the loss to follow-up in our study could lead to the exclusion of a disproportionately high percentage of women who did not intend to breastfeed according to the recommendation. This attrition bias has been described in a Bavarian cohort study by Kohlhuber et al. [42].

In the KUNO Kids study, selection bias is likely because more mothers with a higher education level were inclined to take part in the study. In our population, a clear shift towards higher education could be stated in comparison to the 2004 data of the German population (age cohort 25–44 years) that showed a low education level (CASMIN 1) of 30.4%, a mid-education level (CASMIN 2) of 51.4%, and a high education level (CASMIN 3) of 14.8% [44].

Factors positively influencing breastfeeding behavior in relation to our study’s inclusion bias were the large proportion of highly educated mothers, the above-average social status, the low percentage of critically ill or premature infants, and the study site in a region that previously constituted West Germany, for which Lange et al. had described longer durations of full breastfeeding [8, 32, 45]. Moreover, we excluded mothers with professionally diagnosed mastitis, which is a relevant factor for early breastfeeding cessation [46].

It should be noted, that, for feasibility reasons, we assessed the sub-index health care rather than the entire HLS-EU-Q47 instrument. Nevertheless, Pelikan et al. described a high intercorrelation between the three sub-indices health care, disease prevention, health promotion, and general HL by measuring HL with the HLS-EU instrument [38].

Our study shows a lower proportion of mothers with limited and inadequate HL compared to the general German population (38.8% vs. 44.2%) [17]. This difference may be explained by the age distribution (19–46 years) and the high percentage of highly educated mothers, as both factors are associated with higher HL levels [47].

However, it should be mentioned that HLS-EU items were recorded by means of personal interviews in the presence of a study assistant which may lead to higher HL levels in the context of social desirability.

Nevertheless, the proportion of 38.8% of women with an insufficient HL level seems to be disappointing at first sight. However, one needs to bear in mind that the German Health Literacy Survey using the HLS-EU-Q47 questionnaire yielded a prevalence of limited HL of > 50%, even in people with an intermediate or high level of education [21]. This result as well as our findings needs to be interpreted in consideration of the nature of the HLS-EU questionnaire that asks people to self-assess personal difficulties regarding health issues. Even educated people may report difficulties in finding information on their symptoms or in assessing the reliability of information on one’s illness in the media. An educational reflection on the items presented in the questionnaire may even lead to endorsing more rather than less difficulty.

Obvious strengths of the KUNO-Kids study are its large number of participants of more than 1000 mother–child dyads and its design as a longitudinal cohort study. Furthermore, the Clinic St. Hedwig as Bavaria’s largest perinatal center with patients from urban, suburban, and rural areas provides a good mixture of study participants despite its slightly increased rate of high-risk deliveries [22]. Using items from internationally and nationally validated questionnaires to investigate selected maternal psychosocial characteristics (e.g. CASMIN, HLS-EU, and SF-12) guarantees comparability with other studies.

## Conclusion

The missing association between breastfeeding and HL may be explained by multiple aspects as outlined above. Only a few comparable studies have been published so far. Thus, further studies using comparable measurement methods of HL and breastfeeding are required to objectify relevant factors associated with breastfeeding to develop specific programs for breastfeeding initiation and adherence and to reduce the burden for health-care systems by improving preventive actions.

## Data Availability

The datasets used and/or analyzed for this paper are available from the corresponding author on reasonable request.

## Abbreviation

HL: Health literacy
